# Reducing barriers to the hepatitis C care cascade in prison via point‐of‐care RNA testing: a qualitative exploration of men in prison using an integrated framework

**DOI:** 10.1111/add.16137

**Published:** 2023-02-12

**Authors:** Lise Lafferty, Yumi Sheehan, Amanda Cochrane, Jason Grebely, Andrew R. Lloyd, Carla Treloar

**Affiliations:** ^1^ Centre for Social Research in Health UNSW Sydney Sydney New South Wales Australia; ^2^ The Kirby Institute UNSW Sydney Sydney New South Wales Australia; ^3^ Justice Health and Forensic Mental Health Network NSW Health Matraville New South Wales Australia

**Keywords:** Care cascade, hepatitis C virus, integrated framework, point‐of‐care testing, prisons, qualitative research

## Abstract

**Background and Aims:**

Hepatitis C virus (HCV) is highly prevalent within the prison setting. Although HCV testing and treatment are available within prisons, system barriers can impede progress along the HCV care cascade for those who are incarcerated. The PIVOT intervention used a ‘one‐stop‐shop‘ model (i.e. point‐of‐care HCV RNA testing, Fibroscan‐based liver disease assessment and treatment) at a reception prison in New South Wales, Australia. This analysis sought to understand the role of point‐of‐care HCV RNA testing at intake in reducing barriers to the HCV care cascade within the male prison setting.

**Design and Setting:**

Qualitative analysis using semi‐structured interviews in a reception prison in Australia.

**Participants:**

Twenty‐four men enrolled in the PIVOT study; all participants had undergone HCV point‐of‐care testing in the intervention arm.

**Measurements:**

Høj's Integrated Framework informed this analysis.

**Findings:**

Participants widely expressed the view that point‐of‐care HCV RNA testing on entry was beneficial for care engagement. Point‐of‐care testing was perceived as timely (compared with standard pathology) and reduced opportunities for adjudication by correctional officers due to fewer clinic visits for testing and results. Adoption of routine opt‐out testing at prison intake was regarded as an important strategy for normalising HCV testing (and likely to increase pathways to treatment uptake) and fostered patient candidacy (i.e. self‐perceived eligibility to access care).

**Conclusion:**

Twenty‐four men in prison in New South Wales, Australia, who underwent opt‐out point‐of‐care HCV RNA testing on entry into prison, widely supported the programme as a means of overcoming barriers to HCV testing and treatment in the prison setting, as well as providing public health benefits through early detection of HCV infection among people entering into custody.

## INTRODUCTION

Hepatitis C virus (HCV) is a global public health threat, with 58 million people estimated to be infected [1, 2]. HCV is predominantly transmitted through shared unsterile injecting equipment [3]. Because of the criminalization of injecting drug use, and subsequent widespread incarceration of people who inject drugs, the HCV burden is disproportionately borne by people in prison (15% antibody prevalence globally) [4]. The World Health Organization has set targets to eliminate HCV as a global public health threat by 2030 [5], with prisons identified as a key setting [6, 7, 8].

A period of imprisonment has widely been regarded as an opportune time to engage people in HCV care [9]. Among people engaged in the criminal justice system, the prison setting has been regarded as potentially easier to navigate than community‐based models of care, which can include multiple separate visits to health‐care practitioners, pathology providers and pharmacies (each often requiring travel time) amid other competing priorities [10, 11]. Although deemed more accessible, HCV testing and treatment in the prison setting also has significant barriers. The frequent transfers between prisons and release to the community after very short stays commonly disrupt navigation of the care cascade, with standard pathology results sometimes taking longer for a patient to receive than they are incarcerated [12]. Decentralised, nurse‐led models of care with task transfer from specialist physicians are effective in the prison setting [13, 14], but the timeliness of testing and treatment remains a barrier to link to care for those with shorter sentences. Point‐of‐care HCV RNA testing has been shown to enhance the timeliness of testing and treatment in the community [15, 16, 17], with preliminary evidence for people who are incarcerated [12, 18]. Point‐of‐care HCV testing has been gaining momentum as a timely diagnostic tool in the prison setting. This technology has been used in prisons in several countries, including Australia [19], Canada [20], England [18], Iran [21] and Thailand [22]. However, most of these examples use point‐of‐care antibody testing with provision of reflexive RNA testing.

In a systematic review of harm reduction in European prisons, ~84% of prisons offer hepatitis C testing in the prison setting [23]. However, most of these are delivered under a de facto ‘opt in’ model, whereby people in prison must request testing or have it recommended by a doctor [23]. Only the United Kingdom (UK) provides an opt‐out HCV screening model within prisons, and only nine of 25 countries identified people in prison as a high risk population target for HCV testing [23].

An integrated framework to inform research and practice in HCV care for people who inject drugs was developed in 2019 [24]. The integrated framework was developed to ‘understand, investigate and intervene on modifiable barriers and facilitators to HCV care’ [24] (p. 18). The framework outlines components exploring engagement in the care cascade, including consideration of the patient at the core of these elements. The integrated framework explores identification of patient candidacy (do prospective patients view themselves as viable patients worthy of care), knowledge of care services, navigation of services, service permeability, risk of adjudication and offers and resistance (for treatment) [24]. Point‐of‐care HCV RNA testing has been shown to be acceptable among people in prison [20, 25, 26]. However, identification of the ways in which people entering into prison perceive point‐of‐care testing as a mechanism for HCV care engagement has not been comprehensively explored. Using the integrated framework, this qualitative analysis seeks to identify the ways in which point‐of‐care HCV RNA testing can enhance pathways to HCV care among people entering into prison.

## METHODS

The PIVOT study implemented a ‘one‐stop‐shop’ model incorporating fingerstick point‐of‐care HCV RNA testing (which provided diagnostic results within one hour), fibro‐elastography (Fibroscan; EchoSens Paris), nurse‐led clinical assessment and fast‐tracked direct‐acting antiviral (DAA) prescription for those with a positive HCV RNA test (generally provided the same day, with treatment typically commencing by day six) within a reception prison (that is receiving those newly incarcerated from the community) in New South Wales (NSW), Australia. PIVOT participants were engaged in the ‘one‐stop‐shop’ inclusive of point‐of‐care HCV RNA testing generally within two weeks of prison entry.

Interviews were undertaken with 24 men at a reception prison in NSW, Australia, who had participated in the intervention arm of the PIVOT study (i.e. interviewed after undergoing fingerstick point‐of‐care HCV RNA testing). Interviews were completed between November 2020 and April 2021.

The interview guide was developed by L.L. and Y.S. Interview topics included knowledge of, and experiences with, HCV testing and treatment services in prison, timing of HCV testing in prison, attitudes toward the ‘one‐stop‐shop’ intervention model and acceptability of point‐of‐care HCV RNA testing. Participant demographics were collected before interview commencement.

Potential participants were approached by the study nurse following participation in the intervention arm of the PIVOT trial. Participants were purposively recruited based on history of injecting drug use (IDU) (and no history) and history of venepuncture versus no HCV testing to understand acceptability of HCV point‐of‐care testing with diverse risk profiles and testing experiences. Acceptability of HCV RNA point‐of‐care testing has been found to be acceptable among people with and without histories of injecting drug use and prior HCV testing [25]. Everyone approached for an interview agreed to participate. The study nurse (A.C.) conducted all interviews, with mentorship provided by an experienced prison health researcher (L.L.). Interviews were conducted face‐to‐face in either an interview room or a clinic room with only the interviewer and interviewee present (i.e. no prison officers were present during interviews). Participants were remunerated with AU$10 into their prison bank account. Participant remuneration is standard practice for prison‐based HCV‐focused research in New South Wales correctional centres (see, e.g. previous studies) [27, 28, 29].

Interviews were audio recorded, transcribed verbatim and proofed for accuracy. De‐identified transcripts were then uploaded to NVivo12 qualitative software. A coding memo was developed among authors and informed by the interview guide. Descriptive summaries of each of the nodes were produced and discussed among the authors. A second round of coding was conducted using the integrated framework [24]. Although the framework was specifically designed with consideration of the unique health needs of people who inject drugs, we have applied it here with data from people who are in prison, inclusive of both people with and without histories of injecting drug use. Situated across three ecological domains (individual, service environment and meso‐/macro‐level context), this framework identifies and uses six components, which comprise access to HCV care. These include (1) identification of candidacy (i.e. whether people self‐identify as being eligible for accessing care); (2) navigation of services; (3) service permeability; (4) appearance at services; (5) adjudication by service providers; and (6) offers and resistance. Definitions for each are provided in the Results under relevant subheadings.

Four human research ethics committees provided approval for this qualitative sub‐study: Justice Health and Forensic Mental Health Network (Justice Health NSW) (G975/18); Aboriginal Health and Medical Research Council (1475/19); Corrective Services NSW (date approved: 30 November 2020); and UNSW Sydney (HC1475/19). Pseudonyms are used alongside participant quotes.

## RESULTS

All participants were men who were newly incarcerated and awaiting sentencing (i.e. remandees), with a median age of 30. Participants included men with a history of injecting drug use (*n* = 14), and with (*n* = 14) and without (*n* = 10) prior experience of HCV testing via venepuncture. No participants had previously undergone point‐of‐care HCV RNA fingerstick testing before enrolment in the PIVOT study. A majority (*n* = 14) of participants identified as First Nations. Seven participants received a positive HCV RNA result following point‐of‐care HCV RNA testing within the PIVOT study.

### Identification of candidacy

Identification of candidacy refers to a person's capacity to self‐identify as eligible for receiving health‐care, as well as identification of health need requiring care [24]. All participants were invited to participate in an interview following participation in point‐of‐care HCV RNA testing. Consequently, notions of patient candidacy among PIVOT participants may have been less pronounced, as eligibility for HCV care engagement was offered to all people shortly after they entered the reception prison, in what was ultimately a de facto opt‐out testing model. However, depictions of patient candidacy were apparent in the data. For some, testing was a self‐imposed routine part of their entry into prison, with a smaller number of participants reporting also previously seeking testing before release from an earlier imprisonment. These responses are reflective, and indicative, of the ‘revolving door’ of incarceration.

I don't know how long I'm going to be here, so I'm glad I got that over and done with, so, and I know it's a worry off my shoulders that I'm clear of it. (Jorge, IDU)

Considerations regarding patient candidacy, dependent on perceptions of sentencing status (i.e. sentenced vs awaiting sentencing), were explored with participants. Participants held strong views that all people in prison should be eligible to participate in HCV care, irrespective of their remanded versus sentenced status.

**Okay, would it make a difference if you were sentenced or still remanded?** No, wouldn't make a difference. **Why is that?** Because it doesn't really matter. I don't know how to explain that one, like you are still in custody, it doesn't really matter you know. (Dwayne, No IDU)

‘I don't want to be tested for hep C, because I'm on remand’, that sounds stupid. (Malcolm, IDU)

### Navigation of services

Navigation of services refers to ‘the capacity to reach appropriate services’ and requires ‘an awareness of the suitable sources of care’ [24] (p. 14). Short incarceration periods can inhibit HCV care engagement via traditional testing pathways because of the often lengthy (several weeks) wait for pathology results [19]. Participants identified some of the systemic barriers to HCV care engagement in the prison setting via standard care, particularly the disrupted care cascade resulting from transfers within the prison system and release to freedom (sometimes resulting in a cycle of re‐identification of patient candidacy). Point‐of‐care HCV RNA testing was described as an enabler to service navigation along the care cascade largely because of its ability to deliver timely results.

I wanted to get a blood test and all that sort of stuff, but it takes so long and then I'm out before then. **So short sentences, but moved around a bit or what?** Just short sentences. (Bryan, No IDU)

For the first time in 5 years, I'm actually progressing through it and I'm not just getting blood taken and getting told that we can't … ‘even though we've taken all the blood and we've got every bit of paperwork we can possibly take off you, we still can't give you treatment, because we can't get a [genotype]’ [referring to previous requirements for antiviral treatment]. (Brendan, IDU)

### Service permeability

Service permeability refers to the ease with which patients can access a service, including explicit and implicit gatekeeping or ways in which services can impose barriers, which potential patients may encounter when attempting to access health‐care [24]. Service permeability was less evident within the data as participants had been actively engaged to participate in the PIVOT intervention—that is, the nurse actively engaged people through explaining the fingerstick blood collection process (rather than venepuncture) and delivery of same‐day results, rather than a more passive recruitment process. Regarding the standard care pathway for testing in the prisons, most participants described they were aware they could ‘put a bluey in’ (a special form for requesting a blood borne virus test) or ask a nurse or officer to book them in for an HCV test.

Gatekeepers to health‐care access are regarded within service permeability as part of the integrated framework [24]. Consequently, notions of prison personnel as potential gatekeepers were explored. People in prison, particularly those in prisons with higher security classifications, rely on officers for movement within prison—such as to the clinic to see a nurse. Participants perceived correctional officers as generally supportive of HCV testing in prison, rather than a barrier—or gatekeeper—to HCV care. Occupational health and safety concerns were widely regarded as the primary motivator of officer support for HCV testing and treatment of people in prison. Of note, the non‐judgemental care of prison nurses—particularly HCV nurses—is presented later within ‘Adjudication’.

**Do you think the officers think [the one‐stop‐shop model is] good, like a good thing for us to have?** Yes, because they would be worried about getting like pricked or something you know what I mean. […] If everyone is getting tested […] they wouldn't have to come to work and worry about getting it, you know what I mean? (Antoine, IDU)

### Appearance at services

Appearance at services focuses on the patient's capacity to articulate their health needs when presenting at services [24]. Given that participants were all offered, and participated in, point‐of‐care HCV RNA testing, the need for participants to articulate their health needs was circumvented.

### Adjudication

Adjudication ‘refers to the judgements and decisions of health professionals that allow or inhibit continued progression through the HCV cascade’ [24] (p. 16). Given the scrutiny of the actions and movements of individuals within the prison environment, people accessing HCV care within the prison setting may encounter judgement from different groups as they attempt to progress along the care pathway [30], including from peers, health‐care providers and correctional officers.

Concerns, or anticipations, of adjudication within community health settings were apparent. This encompassed a sense of vulnerability of being perceived as a ‘junkie’ by a participant's regular doctor (Brendan, IDU). Garth contrasts the stigma he encounters at a community clinic compared with the acceptance and non‐judgemental care he receives from the prison nurse.

It's part of your [prison nurse] job to deal with it [hep C] you know what I mean? **Yeah, awesome.** Part of your job not to criticise us for having hep C or stuff like that, like if I'm out [in the community] and I go to a clinic, I feel like they are criticising me whether they are not or they are, but like either way, that's just the way I feel so I won't even bother. **Why do you feel that it's more of a judgment thing outside, compared to inside?** Because you deal with it more on the inside. (Garth, IDU)

### Offers and resistance

Offers and resistance acknowledges that ‘non‐utilisation of services does not necessarily reflect non‐offer’ and that people may decline offers or treatment for a multitude of reasons [24] (p. 16). Participants described being agreeable to offers of HCV testing. George explains below, after having never previously been offered HCV testing, he was satisfied with the process of the offer to participate and undergo testing within the PIVOT study. Again, as depicted within these quotes, it is the non‐judgemental approach of prison health staff that facilitated care engagement among people entering into prison to accept the offer of point‐of‐care HCV RNA testing.

**So what did you like about the way that we did it?** Just the way youse come and approached us and talked to us about it and how everything was worked out and that [the treatment] had a 95% success rate and you know hearing that is like good news you know what I mean. (George, No IDU)

With regards to why participants accepted the point‐of‐care HCV RNA test (identification of candidacy), participants were asked to reflect on when the best time to be offered an HCV test (specifically, a point‐of‐care HCV RNA test) would be, producing several recommendations for testing to be offered at prison intake. This contrasted prior assumptions that people entering into prison may feel overwhelmed by their circumstances and consequently reluctant to engage in HCV care [31]. There were several recommendations made by participants to make testing at entry a mandatory or opt‐out programme, thereby connecting people with treatment, and positioning HCV testing as a ‘normal thing’ (Monte, IDU) at prison entry.

I reckon the beginning would be better than any other time, because you come into custody, get tested, see if you've got it straight away and you could go for early treatment like straight away too. (Michael, No IDU)

Participants also identified that point‐of‐care HCV RNA testing at entry, with fast‐tracked access to treatment, could aid in reducing incidence and prevalence within the prison (and thereby facilitating elimination goals).

Well, you would be better off getting tested when you first come in wouldn't it, because then it won't spread through the jail would it? That would be the best time. (Trent, No IDU)

Less favourable times to be offered testing were also raised. These responses reflected on experiences associated with lengthier incarceration, such as times when people may be experiencing ongoing isolation from family and loved ones. Although Cory describes relying on support from family regarding HCV treatment, this contrasts with responses from other participants who indicated preference for HCV care engagement in prison as it afforded confidentiality from family.

When you haven't had money on your phone in two weeks [laughs] and you just want to make a call and tell your family you love them. […] Contact is very important because like no one in here's got your back for anything let alone finding out you've got hep C. It's good to know that your family like care about you and they can support you even through the phone. (Cory, IDU)

**Is there a particularly bad time to be offering hep C testing?** Christmas. For inmates, any time in the last period of the year, yeah, don't go near people, they're not interested. Their brains aren't with it, no. At the start of the year would be good for inmates, because their mindset is into their jail routine, they'd be heaps more happy, they would be more pleasant toward you and they're listening. (Alec, IDU)

## DISCUSSION

Informed by an integrated framework for HCV care engagement, this analysis identified the ways in which point‐of‐care HCV RNA testing within the prison setting can enhance engagement in the HCV care cascade. Although identification of candidacy and navigation of services were facilitated through participation within the PIVOT study, participants identified patient candidacy would likely be maintained irrespective of sentencing status. Point‐of‐care HCV RNA testing was viewed as a mechanism to overcome barriers to service navigation, and subsequently HCV care engagement, with participants regarding the delivery of same‐day results as enhancing care pathways, particularly in the prison setting. Participants viewed correctional officers as amenable to supporting HCV care engagement (service permeability). Notions of adjudication were apparent, with prison nurses regarded as less likely to impose judgement than community clinicians. Opt‐out point‐of‐care HCV RNA testing at prison entry was regarded as important for personal knowledge of HCV status and, more broadly, as having the potential to foster public health benefits through early detection and treatment of people entering prison with HCV.

The current standard care process for HCV testing in prison is that people who are deemed high risk for blood borne viruses, such as people who report injecting drug use, will be referred for testing—which requires identification of candidacy. Within this qualitative sub‐study, considerations of patient candidacy were broadly overlooked by participants. Albeit participants had all been offered HCV testing, many reflected on why they agreed to be tested, with responses suggestive of ability to identify candidacy and access to care within the prison setting. This ease of self‐identification is perhaps reflective of the changing landscape of HCV treatment eligibility amid the direct‐acting antivirals (DAA) era. Substantial restrictions to HCV treatment were enacted throughout the interferon era, including drug use abstinence, whereby potential HCV treatment patients were expected to negotiate and advocate for their care needs [32]. With the emergence of data demonstrating that interferon‐based HCV treatment was safe and effective among people who inject drugs [33], restrictions based on fibrosis stage and recent drug and/or alcohol use were then placed on the reimbursement of these therapies in many countries including Canada [34], Europe [35] and the United States [36]. This led to ongoing challenges regarding self‐perception of patient candidacy/self‐identification. However, decreasing DAA prices and data on the effectiveness of HCV DAA therapy among people who use drugs [37] and low rates of HCV reinfection [38] helped to facilitate policy change to remove many of the historical barriers to patient candidacy for HCV care engagement (e.g. drug use abstinence) [35, 39]. In the United States, the removal of Medicaid restrictions has facilitated an increase in uptake of DAA treatment [40]. Our findings suggest that the advent of DAAs coupled with subsequent policy changes including universal access of DAAs for people in prison with chronic HCV have enabled people to be more proactive in self‐identification of their HCV‐related health‐care needs.

Lack of officer knowledge and education relating to HCV risk and transmission have consistently been reported as drivers of stigma in custodial settings [41, 42]. However, participants perceived broad officer support of HCV care engagement, suggesting a reduction in stigma associated with HCV in custodial environments. They also attributed perceived officer support as due to the individual safety and public health gains for the prison if everyone entering into custody were tested and treated for HCV. In light of Dwayne's remarks about lack of knowledge of HCV as a barrier to dialogue, these findings evidence the important role of education to further reduce barriers to HCV care engagement in the prison setting.

A recent systematic review and meta‐analysis reporting on interventions to enhance HCV care engagement concluded that ‘Interventions addressing setting‐specific and population‐specific barriers are likely to be the most effective at improving HCV care’ [43] (p. 440). Initiatives to achieve HCV micro‐elimination in the prison setting have identified opt‐out testing at prison entry as an invaluable component of elimination efforts [23, 44]. Modelling of opt‐out point‐of‐care HCV RNA testing at prison entry has been shown to be cost‐effective [45]. Other research has found that point‐of‐care testing (inclusive of Oraquick, Dried Blood Spot and GeneXpert fingerstick assay) in the prison setting greatly enhances engagement in the HCV care cascade, compared with standard of care [18]. This is because of the reduced systems barriers—often associated with increased need for timely treatment because of movement within the prison as well as short sentences and transfer of care to community [12, 46]. A qualitative study conducted in the United Kingdom found that being offered HCV testing in prison was regarded as a valuable health check [26]. Considerations of timing and recommendations that opt‐out point‐of‐care HCV RNA testing be implemented at prison entry was viewed as enhancing opportunities for HCV care engagement in the prison setting. As Monte indicated, testing at entry would likely normalise HCV care engagement. In combination with other findings, such as perceived judgement or concerns of external adjudication, normalisation of testing would likely further reduce social barriers to HCV testing and would remove the need for people in prison to self‐identify patient candidacy and exert agency to access and attend testing.

This study has limitations. Participants in this study were on remand at the time of participation in the broader PIVOT study. This may have had influence on participants' perceptions of sentencing status with regards to identification of candidacy, as well as timing of being offered testing. However, being held on remand is likely a more vulnerable experience than entering into prison with a sentence because of the multitude of unknowns about one's future. Addressing HCV at this early stage of incarceration was widely viewed as an important time and was seen as manageable, irrespective of other stressors, which a newly incarcerated person may experience. All participants in this study were men and may not reflect the experiences of women. In a survey of prison entrants in Australia, women were twice as likely as men to score in the high to very high range of stress on the Kessler 10 scale [47]. Therefore, it may be that women entering into prison may require additional supports at this critical time or may not feel emotionally equipped to receive a positive HCV result, particularly in the absence of family supports.

The findings presented in this paper suggest that opt‐out point‐of‐care HCV RNA testing on entry into prison could reduce many of the barriers identified by participants. Offering testing to all prison entrants could ensure that people had the opportunity to get tested without requiring them to consider their candidacy. Additionally, opt‐out screening at the point of intake using point‐of‐care testing could ensure patients received their results the same day—likely enabling pathways to treatment before release or prison transfer. Integration of opt‐out testing for all people entering into custody could circumvent the barriers identified in navigation of services and could provide health‐care engagement opportunities to learn about the HCV services available in the prison setting. Last, HCV care could be normalised within environments in which all people were offered HCV testing. Future research relating to the timeliness of point‐of‐care HCV RNA testing at prison entry should consider the role of gender and how experiences of incarceration may differ for men and women, particularly within the context of point‐of‐care HCV RNA testing.

## DECLARATION OF INTERESTS

L.L. has received speaker fees from AbbVie. A.C. has received speaker fees from AbbVie. Y.S. has no conflicts of interest to report. J.G. is a consultant/advisor and has received research grants from AbbVie, Camurus, Cepheid, Gilead Sciences, Hologic, Indivior and Merck/MSD. A.R.L. has received investigator‐initiated research support from Gilead and AbbVie. C.T. has received speaker fees from AbbVie and Gilead Sciences.

## AUTHOR CONTRIBUTIONS

**Lise Lafferty:** Conceptualization; formal analysis; funding acquisition; methodology; writing ‐ original draft. **Yumi Sheehan:** Conceptualization; methodology; project administration; writing ‐ review and editing. **Amanda Cochrane:** Investigation; writing ‐ review and editing. **Jason Grebely:** Conceptualization; funding acquisition; methodology; writing ‐ review and editing. **Andrew Lloyd:** Conceptualization; funding acquisition; methodology; writing ‐ review and editing. **Carla Treloar:** Conceptualization; methodology; writing ‐ review and editing.

## CLINICAL TRIAL REGISTRATION

NCT04809246.
